# 6,12-Bis(hex­yloxy)-5*H*,11*H*-indolo[3,2-*b*]carbazole

**DOI:** 10.1107/S1600536812050611

**Published:** 2012-12-19

**Authors:** Norma Wrobel, Bernhard Witulski, Dieter Schollmeyer, Heiner Detert

**Affiliations:** a University Mainz, Duesbergweg 10-14, 55099 Mainz, Germany; b Laboratoire de Chimie Moléculaire et Thio-organique, UMR 6507, ENSICAEN, 6 Boulevard Maréchal Juin, 14050 Caen, France

## Abstract

The title compound, C_30_H_36_N_2_O_2_, was prepared in a twofold Cadogan cyclization. The mol­ecule is located about a center of inversion. The indolocarbazole skeleton is essentially planar [maximum deviation = 0.028 (2) Å], the C—N bond lengths are nearly identical and the C—C bond lengths of the pyrrole unit are significantly longer than those of the benzene subunits.

## Related literature

For the synthesis and structure of the starting material, see: Wrobel *et al.* (2012 ▶). For the Cadogan reaction, see: Cadogan (1962 ▶, 1969 ▶). For other approaches to Indolocarbazoles, see: Knölker & Reddy (2002 ▶); Katritzky *et al.* (1995 ▶). For electronic properties of indolocarbazoles, see: Hu *et al.* (1999 ▶); Wakim *et al.* (2004 ▶); Nemkovich *et al.* (2009 ▶). For heteroanalogous carbazoles, see: Dassonneville *et al.* (2011 ▶); Nissen & Detert (2011 ▶); Letessier & Detert (2012 ▶); Letessier *et al.* (2012 ▶). For conjugated oligomers see: Detert *et al.* (2010 ▶).
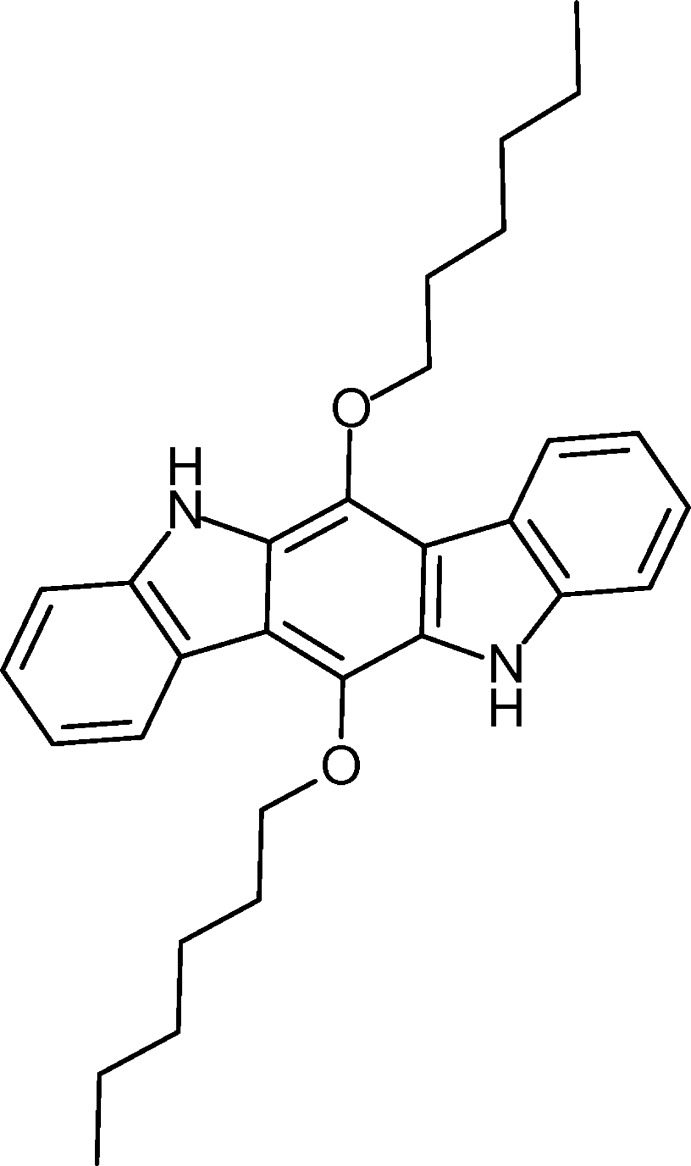


## Experimental

### 

#### Crystal data

C_30_H_36_N_2_O_2_
*M* *_r_* = 456.61
Monoclinic, 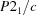

*a* = 13.7136 (4) Å
*b* = 5.5026 (4) Å
*c* = 16.5563 (5) Å
β = 92.665 (3)°
*V* = 1247.99 (10) Å^3^
*Z* = 2
Cu *K*α radiation
μ = 0.59 mm^−1^
*T* = 298 K
0.48 × 0.26 × 0.18 mm

#### Data collection

Enraf–Nonius CAD-4 diffractometer
2466 measured reflections
2363 independent reflections
1993 reflections with *I* > 2σ(*I*)
*R*_int_ = 0.052
3 standard reflections every 60 min intensity decay: 5%

#### Refinement

*R*[*F* ^2^ > 2σ(*F* ^2^)] = 0.057
*wR*(*F* ^2^) = 0.181
*S* = 1.06
2363 reflections
168 parameters
Only H-atom displacement parameters refined
Δρ_max_ = 0.26 e Å^−3^
Δρ_min_ = −0.29 e Å^−3^

### 

Data collection: *CAD-4 Software* (Enraf–Nonius, 1989 ▶); cell refinement: *CAD-4 Software*; data reduction: *CORINC* (Dräger & Gattow, 1971 ▶); program(s) used to solve structure: *SIR97* (Altomare *et al.*, 1999 ▶); program(s) used to refine structure: *SHELXL97* (Sheldrick, 2008 ▶); molecular graphics: *PLATON* (Spek, 2009 ▶); software used to prepare material for publication: *SHELXL97*.

## Supplementary Material

Crystal structure: contains datablock(s) I, global. DOI: 10.1107/S1600536812050611/nc2301sup1.cif

Structure factors: contains datablock(s) I. DOI: 10.1107/S1600536812050611/nc2301Isup2.hkl

Supplementary material file. DOI: 10.1107/S1600536812050611/nc2301Isup3.cml

Additional supplementary materials:  crystallographic information; 3D view; checkCIF report

## Figures and Tables

**Fig. 1.**
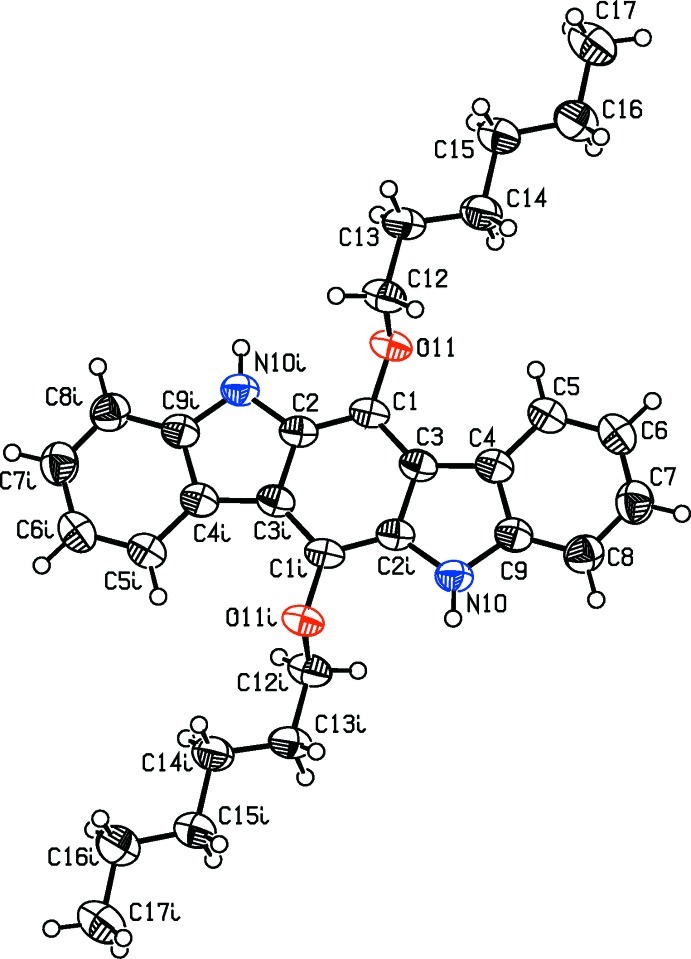
Crystal structure of the title compound with labeling and displacement ellipsoids drawn at the 50% probability level. Symmetry codes: i = 1 - x,1 - y,1 - z.

## supplementary crystallographic information

### Comment

As part of a larger project on the synthesis of carbazoles (Letessier & Detert, 2012) and carbolines (Dassonneville *et al.* 2011; Nissen & Detert, 2011; Letessier *et al.* 2012); indolo-annulated carbazoles were prepared for optoelectronic applications. The title compounds adopts a centrosymmetric geometry. The pentacyclic indolocarbazole framework is essentially planar with maximum deviations of 0.028 (2) Å from the mean plane. The dihedral angle between the mean plane of the aromatic system and and the adjacend *O*-alkyl unit (C3—C1—O1—C12) is -101.5 (2)° and the all-*trans* configured hexyl chain lies in a plane parallel to that of the aromatic system. Whereas the O11—C12—C13—C14 unit adopts a *gauche* conformation (torsion angle = -71.5 (3)°) the tail of the hexyl chain is nearly planar (dihedral angles -171.3 (2)°, 175.0 (2)°, 176.7 (2)°). The C—N bonds in the pyrrole units are nearly identical. The C—C bonds in the pyrrole subunit (C2—C3 = 1.418 (3) Å, C3—C4 1.448 (3) Å, C4—C9 1.406 (3) Å) are significantly longer than those of the benzene units (C4—C5 = 1.402 (3) Å, C5—C6 = 1.383 (3) Å, C6—C7 = 1.386 (3) Å, C7—C8 = 1.385 (3) Å, C8—C9 = 1.392 (3) Å, C1—C2 = 1.388 (3) Å, C1—C3 = 1.395 (3) Å). The hexyloxy chains are interdigitated.

### Experimental

6,12-Dihexyloxyindolo[3,2-*b*]carbazole was prepared from 1,4-dihexyloxy-2,5-bis(2-nitrophenyl)benzene (Wrobel *et al.* 2012) *via* Cadogan cyclization. In a microwave reactor tube 400 mg of the dinitro-compound were mixed with triethyl phosphite (4 ml) and irradiated (300 W, 483 K) for 15 min. The cooled mixture was dissolved in ethyl acetate (50 ml), and the same amount of hydrochloric acid (6 N) was added and the mixture heated for 3 h to reflux. After dilution with water, the product was extracted with dichloromethane (3x), the pooled organic solutions were washed with brine, dried (MgSO_4_), and concentrated. Purification by column chromatography (SiO_2_, petroleum ether/ethyl acetate = 9/1 (*v*/*v*), *R*_f_ = 0.40). Yield: 213 mg (61%) of an off-white solid with m.p. = 422–424 K. Single crystals were obtained by slow evaporation of a solution of the title compound in chloroform/ethanol (5/1).

### Refinement

Hydrogen atoms attached to carbons were placed at calculated positions (methyl H atoms allowed to rotate but not to tip) with C—H = 0.93 Å for aromatic, 0.97 Å for methylene and 0.96 Å for methyl H atoms and were refined in the riding-model approximation with a common isotropic displacement parameters for those H atoms connected to the same C atom. The N—H atom was located in the difference Fourier map and were refined using a riding model additional allowing drifting along the N–H vector.

### Crystal data

**Table:**

C_30_H_36_N_2_O_2_	*F*(000) = 492
*M**_r_* = 456.61	*D*_x_ = 1.215 Mg m^−^^3^
Monoclinic, *P*2_1_/*c*	Melting point: 423 K
Hall symbol: -P 2ybc	Cu *K*α radiation, λ = 1.54178 Å
*a* = 13.7136 (4) Å	Cell parameters from 25 reflections
*b* = 5.5026 (4) Å	θ = 35–52°
*c* = 16.5563 (5) Å	µ = 0.59 mm^−^^1^
β = 92.665 (3)°	*T* = 298 K
*V* = 1247.99 (10) Å^3^	Needle, colourless
*Z* = 2	0.48 × 0.26 × 0.18 mm

### Data collection

**Table:**

Enraf–Nonius CAD-4 diffractometer	*R*_int_ = 0.052
Radiation source: rotating anode	θ_max_ = 70.0°, θ_min_ = 3.2°
Graphite monochromator	*h* = 0→16
ω/2θ scans	*k* = −6→0
2466 measured reflections	*l* = −20→20
2363 independent reflections	3 standard reflections every 60 min
1993 reflections with *I* > 2σ(*I*)	intensity decay: 5%

### Refinement

**Table:**

Refinement on *F*^2^	Secondary atom site location: difference Fourier map
Least-squares matrix: full	Hydrogen site location: difference Fourier map
*R*[*F*^2^ > 2σ(*F*^2^)] = 0.057	Only H-atom displacement parameters refined
*wR*(*F*^2^) = 0.181	*w* = 1/[σ^2^(*F*_o_^2^) + (0.0992*P*)^2^ + 0.4797*P*] where *P* = (*F*_o_^2^ + 2*F*_c_^2^)/3
*S* = 1.06	(Δ/σ)_max_ = 0.001
2363 reflections	Δρ_max_ = 0.26 e Å^−^^3^
168 parameters	Δρ_min_ = −0.29 e Å^−^^3^
0 restraints	Extinction correction: *SHELXL97* (Sheldrick, 2008), Fc^*^=kFc[1+0.001xFc^2^λ^3^/sin(2θ)]^-1/4^
Primary atom site location: structure-invariant direct methods	Extinction coefficient: 0.0083 (12)

### Special details

**Table:**

Experimental. H-NMR (400 MHz, CDCl_3_): 10.94 (s, 2 H, NH), 8.20 (d, J = 7.7 Hz, 2 H), 7.49 (d, J = 8.1 Hz, 2 H), 7.38 (dt, J = 7.6 Hz, JX= 1.2 Hz, 2 H), 7.12 (dt, J = 7.4 Hz, JX= 0.9 Hz, 2 H), 4.25 (t, J = 7.0 Hz, 4 H, OCH_2_), 1.98 (m, 4 H, β-CH_2_), 1.56 - 1.31 (m, 12 H), 0.87 (m, 6 H, CH_3_).C-NMR (75 MHz, CDCl_3_): 140.9 (*s*), 133.7 (*s*), 127.7 (*s*), 125.4 (*d*), 122.0 (*d*), 121.7 (*s*), 118.1 (*d*), 116.4 (*s*), 110.8 (*d*), 72.7 (*t*), 31.3 (*t*), 30.0 (*t*), 25.3 (*t*), 22.2 (*t*), 14.0 (*q*).IR (ATR) 3435, 3292, 2954, 2924, 2909, 2863, 2357, 1916, 1886, 1776, 1615, 1539, 1455, 1403, 1383, 1334, 1298, m1251, 1215, 1149, 1123, 1074, 1049, 1028, 1006, 983, 916 cm^-1^.MS (EI): 456 (59%) [*M*]^+^; 187 (100%) [*M*-2 C_6_H_12_]^+^UV-Vis (dichloromethane): λ = 377 nm (log ε = 3.82); 394 nm (log ε = 3.84); Fluorescence: 407 nm (dichloromethane).Combustion analysis: calc. for C_30_H_36_N_2_O_2_: C: 78.91%, H: 7.95%, N: 6.13%. Found: C: 78.56%, H: 8.04%, N: 6.09%.
Geometry. All e.s.d.'s (except the e.s.d. in the dihedral angle between two l.s. planes) are estimated using the full covariance matrix. The cell e.s.d.'s are taken into account individually in the estimation of e.s.d.'s in distances, angles and torsion angles; correlations between e.s.d.'s in cell parameters are only used when they are defined by crystal symmetry. An approximate (isotropic) treatment of cell e.s.d.'s is used for estimating e.s.d.'s involving l.s. planes.
Refinement. Refinement of *F*^2^ against ALL reflections. The weighted *R*-factor *wR* and goodness of fit *S* are based on *F*^2^, conventional *R*-factors *R* are based on *F*, with *F* set to zero for negative *F*^2^. The threshold expression of *F*^2^ > σ(*F*^2^) is used only for calculating *R*-factors(gt) *etc*. and is not relevant to the choice of reflections for refinement. *R*-factors based on *F*^2^ are statistically about twice as large as those based on *F*, and *R*- factors based on ALL data will be even larger.

### Fractional atomic coordinates and isotropic or equivalent isotropic displacement parameters (Å^2^)

**Table:**

	*x*	*y*	*z*	*U*_iso_*/*U*_eq_	
C1	0.42601 (13)	0.3175 (3)	0.48922 (12)	0.0434 (5)	
C2	0.49063 (13)	0.3189 (3)	0.55632 (12)	0.0433 (5)	
C3	0.43593 (12)	0.5015 (3)	0.43222 (12)	0.0430 (5)	
C4	0.38310 (13)	0.5621 (4)	0.35713 (12)	0.0442 (5)	
C5	0.30437 (14)	0.4564 (4)	0.31300 (13)	0.0501 (5)	
H5	0.2752	0.3154	0.3314	0.050 (6)*	
C6	0.27074 (16)	0.5650 (5)	0.24173 (14)	0.0587 (6)	
H6	0.2182	0.4969	0.2122	0.068 (7)*	
C7	0.31424 (17)	0.7740 (5)	0.21368 (14)	0.0607 (6)	
H7	0.2902	0.8433	0.1655	0.078 (8)*	
C8	0.39253 (16)	0.8825 (4)	0.25545 (13)	0.0545 (6)	
H8	0.4216	1.0223	0.2361	0.060 (7)*	
C9	0.42607 (13)	0.7741 (4)	0.32748 (12)	0.0449 (5)	
N10	0.50337 (11)	0.8415 (3)	0.37876 (10)	0.0463 (5)	
H10	0.5328 (10)	0.985 (5)	0.37714 (11)	0.067 (7)*	
O11	0.35653 (9)	0.1369 (2)	0.47790 (8)	0.0478 (4)	
C12	0.27228 (15)	0.1745 (4)	0.52366 (14)	0.0552 (6)	
H12A	0.2391	0.3220	0.5058	0.071 (5)*	
H12B	0.2914	0.1920	0.5805	0.071 (5)*	
C13	0.20475 (16)	−0.0403 (5)	0.51188 (14)	0.0613 (6)	
H13A	0.2423	−0.1877	0.5216	0.086 (6)*	
H13B	0.1559	−0.0326	0.5523	0.086 (6)*	
C14	0.15331 (16)	−0.0584 (4)	0.42985 (14)	0.0577 (6)	
H14A	0.2009	−0.0930	0.3899	0.071 (5)*	
H14B	0.1233	0.0968	0.4163	0.071 (5)*	
C15	0.07519 (17)	−0.2551 (5)	0.42592 (15)	0.0627 (6)	
H15A	0.1065	−0.4114	0.4352	0.088 (7)*	
H15B	0.0315	−0.2283	0.4694	0.088 (7)*	
C16	0.01583 (19)	−0.2668 (6)	0.34785 (17)	0.0729 (8)	
H16A	0.0588	−0.3037	0.3046	0.131 (10)*	
H16B	−0.0128	−0.1083	0.3369	0.131 (10)*	
C17	−0.0645 (2)	−0.4534 (6)	0.34732 (18)	0.0794 (8)	
H17A	−0.1012	−0.4470	0.2965	0.114 (7)*	
H17B	−0.1070	−0.4198	0.3905	0.114 (7)*	
H17C	−0.0366	−0.6123	0.3546	0.114 (7)*	

### Atomic displacement parameters (Å^2^)

**Table:**

	*U*^11^	*U*^22^	*U*^33^	*U*^12^	*U*^13^	*U*^23^
C1	0.0352 (9)	0.0377 (10)	0.0578 (11)	−0.0060 (7)	0.0076 (8)	−0.0050 (8)
C2	0.0374 (9)	0.0390 (10)	0.0539 (11)	−0.0025 (8)	0.0068 (8)	−0.0003 (8)
C3	0.0357 (9)	0.0399 (10)	0.0537 (11)	−0.0016 (8)	0.0051 (8)	−0.0040 (8)
C4	0.0387 (9)	0.0418 (10)	0.0526 (11)	0.0013 (7)	0.0058 (8)	−0.0055 (8)
C5	0.0436 (10)	0.0495 (11)	0.0571 (12)	−0.0043 (9)	0.0004 (9)	−0.0048 (9)
C6	0.0502 (12)	0.0659 (14)	0.0593 (13)	−0.0025 (10)	−0.0070 (10)	−0.0077 (11)
C7	0.0572 (12)	0.0666 (15)	0.0577 (13)	0.0078 (11)	−0.0028 (10)	0.0035 (11)
C8	0.0527 (12)	0.0503 (12)	0.0608 (13)	0.0037 (9)	0.0051 (9)	0.0057 (10)
C9	0.0394 (9)	0.0425 (10)	0.0531 (11)	0.0015 (8)	0.0052 (8)	−0.0030 (8)
N10	0.0436 (9)	0.0399 (9)	0.0554 (10)	−0.0058 (7)	0.0031 (7)	0.0018 (7)
O11	0.0406 (7)	0.0415 (8)	0.0618 (9)	−0.0100 (6)	0.0071 (6)	−0.0094 (6)
C12	0.0434 (11)	0.0580 (13)	0.0650 (13)	−0.0121 (10)	0.0104 (9)	−0.0111 (10)
C13	0.0526 (12)	0.0632 (14)	0.0683 (14)	−0.0213 (11)	0.0049 (10)	0.0022 (11)
C14	0.0501 (12)	0.0532 (13)	0.0697 (14)	−0.0101 (10)	0.0003 (10)	0.0028 (10)
C15	0.0562 (13)	0.0605 (14)	0.0710 (15)	−0.0148 (11)	−0.0016 (11)	0.0019 (11)
C16	0.0620 (14)	0.0834 (18)	0.0731 (16)	−0.0141 (13)	−0.0005 (12)	−0.0021 (14)
C17	0.0616 (14)	0.087 (2)	0.0886 (19)	−0.0149 (14)	−0.0026 (13)	−0.0178 (16)

### Geometric parameters (Å, º)

**Table:**

C1—O11	1.383 (2)	O11—C12	1.426 (2)
C1—C2	1.388 (3)	C12—C13	1.508 (3)
C1—C3	1.395 (3)	C12—H12A	0.9700
C2—N10^i^	1.390 (3)	C12—H12B	0.9700
C2—C3^i^	1.418 (3)	C13—C14	1.504 (3)
C3—C2^i^	1.418 (3)	C13—H13A	0.9700
C3—C4	1.448 (3)	C13—H13B	0.9700
C4—C5	1.402 (3)	C14—C15	1.522 (3)
C4—C9	1.406 (3)	C14—H14A	0.9700
C5—C6	1.383 (3)	C14—H14B	0.9700
C5—H5	0.9300	C15—C16	1.496 (4)
C6—C7	1.386 (3)	C15—H15A	0.9700
C6—H6	0.9300	C15—H15B	0.9700
C7—C8	1.385 (3)	C16—C17	1.506 (4)
C7—H7	0.9300	C16—H16A	0.9700
C8—C9	1.392 (3)	C16—H16B	0.9700
C8—H8	0.9300	C17—H17A	0.9600
C9—N10	1.378 (2)	C17—H17B	0.9600
N10—C2^i^	1.390 (3)	C17—H17C	0.9600
N10—H10	0.89 (3)		
			
O11—C1—C2	121.48 (17)	O11—C12—H12B	109.9
O11—C1—C3	121.19 (18)	C13—C12—H12B	109.9
C2—C1—C3	117.30 (17)	H12A—C12—H12B	108.3
C1—C2—N10^i^	128.88 (17)	C14—C13—C12	115.3 (2)
C1—C2—C3^i^	122.28 (18)	C14—C13—H13A	108.4
N10^i^—C2—C3^i^	108.84 (17)	C12—C13—H13A	108.4
C1—C3—C2^i^	120.43 (18)	C14—C13—H13B	108.4
C1—C3—C4	133.40 (17)	C12—C13—H13B	108.4
C2^i^—C3—C4	106.16 (16)	H13A—C13—H13B	107.5
C5—C4—C9	119.18 (19)	C13—C14—C15	112.67 (19)
C5—C4—C3	133.98 (19)	C13—C14—H14A	109.1
C9—C4—C3	106.84 (16)	C15—C14—H14A	109.1
C6—C5—C4	118.9 (2)	C13—C14—H14B	109.1
C6—C5—H5	120.5	C15—C14—H14B	109.1
C4—C5—H5	120.5	H14A—C14—H14B	107.8
C5—C6—C7	120.9 (2)	C16—C15—C14	114.9 (2)
C5—C6—H6	119.6	C16—C15—H15A	108.5
C7—C6—H6	119.6	C14—C15—H15A	108.5
C8—C7—C6	121.7 (2)	C16—C15—H15B	108.5
C8—C7—H7	119.1	C14—C15—H15B	108.5
C6—C7—H7	119.1	H15A—C15—H15B	107.5
C7—C8—C9	117.4 (2)	C15—C16—C17	113.8 (2)
C7—C8—H8	121.3	C15—C16—H16A	108.8
C9—C8—H8	121.3	C17—C16—H16A	108.8
N10—C9—C8	128.81 (19)	C15—C16—H16B	108.8
N10—C9—C4	109.33 (17)	C17—C16—H16B	108.8
C8—C9—C4	121.83 (19)	H16A—C16—H16B	107.7
C9—N10—C2^i^	108.77 (16)	C16—C17—H17A	109.5
C9—N10—H10	123.97 (11)	C16—C17—H17B	109.5
C2^i^—N10—H10	125.27 (11)	H17A—C17—H17B	109.5
C1—O11—C12	113.19 (14)	C16—C17—H17C	109.5
O11—C12—C13	108.99 (18)	H17A—C17—H17C	109.5
O11—C12—H12A	109.9	H17B—C17—H17C	109.5
C13—C12—H12A	109.9		
			
O11—C1—C2—N10^i^	−1.9 (3)	C6—C7—C8—C9	−0.3 (3)
C3—C1—C2—N10^i^	−179.96 (18)	C7—C8—C9—N10	178.2 (2)
O11—C1—C2—C3^i^	178.17 (16)	C7—C8—C9—C4	0.3 (3)
C3—C1—C2—C3^i^	0.2 (3)	C5—C4—C9—N10	−178.17 (17)
O11—C1—C3—C2^i^	−178.18 (16)	C3—C4—C9—N10	1.7 (2)
C2—C1—C3—C2^i^	−0.2 (3)	C5—C4—C9—C8	0.1 (3)
O11—C1—C3—C4	3.1 (3)	C3—C4—C9—C8	179.96 (17)
C2—C1—C3—C4	−178.89 (19)	C8—C9—N10—C2^i^	179.61 (19)
C1—C3—C4—C5	−1.8 (4)	C4—C9—N10—C2^i^	−2.3 (2)
C2^i^—C3—C4—C5	179.4 (2)	C2—C1—O11—C12	80.5 (2)
C1—C3—C4—C9	178.37 (19)	C3—C1—O11—C12	−101.5 (2)
C2^i^—C3—C4—C9	−0.5 (2)	C1—O11—C12—C13	−175.89 (17)
C9—C4—C5—C6	−0.5 (3)	O11—C12—C13—C14	−71.5 (3)
C3—C4—C5—C6	179.7 (2)	C12—C13—C14—C15	−171.3 (2)
C4—C5—C6—C7	0.5 (3)	C13—C14—C15—C16	175.0 (2)
C5—C6—C7—C8	−0.1 (4)	C14—C15—C16—C17	−176.7 (2)

Symmetry code: (i) −*x*+1, −*y*+1, −*z*+1.

## References

[bb1] Altomare, A., Burla, M. C., Camalli, M., Cascarano, G. L., Giacovazzo, C., Guagliardi, A., Moliterni, A. G. G., Polidori, G. & Spagna, R. (1999). *J. Appl. Cryst.* **32**, 115–119.

[bb2] Cadogan, J. I. G. (1962). *Q. Rev.* **16**, 208–239.

[bb3] Cadogan, J. I. G. (1969). *Synthesis*, pp. 11–17.

[bb4] Dassonneville, B., Witulski, B. & Detert, H. (2011). *Eur. J. Org. Chem.* pp. 2836–2844.

[bb5] Detert, H., Lehmann, M. & Meier, H. (2010). Materials, **3**, 3218–3330.

[bb6] Dräger, M. & Gattow, G. (1971). *Acta Chem. Scand.* **25**, 761–762.

[bb7] Enraf–Nonius (1989). *CAD-4 Software* Enraf–Nonius, Delft, The Netherlands.

[bb8] Hu, N.-X., Xie, S., Popovic, Z., Ong, B. & Hor, A.-M. (1999). *J. Am. Chem. Soc* **121**, 5097–5098.

[bb9] Katritzky, A. R., Li, J. & Stevens, C. V. (1995). *J. Org. Chem.* **60**, 3401–3404.

[bb10] Knölker, H.-J. & Reddy, K. R. (2002). *Chem. Rev.* **39**, 6521–6527.

[bb11] Letessier, J. & Detert, H. (2012). *Synthesis*, **44**, 290–296.

[bb12] Letessier, J., Detert, H., Götz, K. & Opatz, T. (2012). *Synthesis*, **44**, 747–754.

[bb13] Nemkovich, N. A., Kruchenok, Yu. V., Sobchuk, A. N., Detert, H., Wrobel, N. & Chernyavskii, E. A. (2009). *Opt. Spectrosc.* **107**, 275–281.

[bb14] Nissen, F. & Detert, H. (2011). *Eur. J. Org. Chem.* pp. 2845–2854.

[bb15] Sheldrick, G. M. (2008). *Acta Cryst.* A**64**, 112–122. 10.1107/S0108767307043930 18156677

[bb16] Spek, A. L. (2009). *Acta Cryst.* D**65**, 148–155. 10.1107/S090744490804362X PMC2631630 19171970

[bb17] Wakim, S., Bouchard, J., Simard, M., Drolet, N., Tao, Y. & Leclerc, M. (2004). *Chem. Mater.* **16**, 4386–4388.

[bb18] Wrobel, N., Schollmeyer, D. & Detert, H. (2012). *Acta Cryst.* E**68**, o1022. 10.1107/S1600536812009944 PMC3343986 22589895

